# Inhibition of Carrageenan-Induced Acute Inflammation in Mice by the *Microgramma vacciniifolia* Frond Lectin (MvFL)

**DOI:** 10.3390/polym14081609

**Published:** 2022-04-15

**Authors:** Leydianne Leite de Siqueira Patriota, Dalila de Brito Marques Ramos, Mariana Gama e Silva, Angela Caroline Lima Amorim dos Santos, Yasmym Araújo Silva, Patrícia Maria Guedes Paiva, Emmanuel Viana Pontual, Lidiane Pereira de Albuquerque, Rosemairy Luciane Mendes, Thiago Henrique Napoleão

**Affiliations:** 1 Departamento de Bioquímica, Centro de Biociências, Universidade Federal de Pernambuco, Recife 50670-901, Pernambuco, Brazil; leydianne.patriota@ufpe.br (L.L.d.S.P.); patricia.paiva@ufpe.br (P.M.G.P.); 2 Campus Amilcar Ferreira Sobral, Universidade Federal do Piauí, Floriano 64808-605, Piauí, Brazil; dalila.ramos@hotmail.com; 3 Laboratório de Oncologia Experimental, Universidade Federal do Vale do São Francisco, Petrolina 56306-385, Pernambuco, Brazil; marianagama91@hotmail.com (M.G.e.S.); santos.angelacontato@gmail.com (A.C.L.A.d.S.); yasmymsilva@hotmail.com (Y.A.S.); rosemairy.mendes@univasf.edu.br (R.L.M.); 4 Departamento de Morfologia e Fisiologia Animal, Universidade Federal Rural de Pernambuco, Recife 52171-900, Pernambuco, Brazil; emmanuel.pontual@ufrpe.br; 5 Departamento de Bioquímica e Farmacologia, Universidade Federal do Piauí, Teresina 64049-550, Piauí, Brazil; lidianealbuquerque@ufpi.edu.br

**Keywords:** plant lectin, *Microgramma vacciniifolia*, inflammation, cytokines, anti-inflammatory agents, carrageenan

## Abstract

Most anti-inflammatory drugs used nowadays have an excessive cost and their prolonged use has been connected with several injurious effects. Thus, the search for new anti-inflammatory agents is increasing. Lectins are carbohydrate-interacting proteins that can modulate immune response and the release of inflammation mediators. The *Microgramma vacciniifolia* frond lectin (MvFL) was previously reported to be an immunomodulatory agent in vitro. This work aimed to evaluate the effects of MvFL on the in vivo inflammatory status in the carrageenan-induced peritonitis and paw edema, using female Swiss mice. The animals were pretreated intraperitoneally with MvFL (5 and 10 mg/kg). In the peritonitis assay, the total and differential migration of white blood cells was evaluated, as well as the levels of cytokines, nitric oxide (NO), and total proteins in the peritoneal fluid. In the paw edema evaluation, the paw volume was measured in the early (from 30 min–2 h) and late (3–4 h) phases of edema formation. MvFL (5 and 10 mg/kg) was efficient in reducing neutrophil infiltration, pro-inflammatory cytokines (IL-6, IL-17, and TNF-α), NO, and protein content in the peritoneal fluid. It also repressed the edema formation in the late phase of the assay. In conclusion, MvFL showed inhibitory effects in in vivo acute inflammation, which encouraged future studies exploiting its immunomodulatory ability.

## 1. Introduction

Inflammatory responses have a crucial role in the immune defense towards injuries, pathogens, and toxins [1]. However, inflammatory dysregulation participates in several illnesses, including diabetes, cardiovascular diseases, cancer, and autoimmune diseases [2]. Nowadays, the most used anti-inflammatory drugs have an excessive cost, and their lengthened use has been connected with adverse effects such as adrenal atrophy, cataracts, euphoria, glaucoma, osteoporosis, overthrow of response to infection or injury, and peptic ulcers. In this way, the pursuit of new anti-inflammatory agents, including natural products from plants, is increasing, aiming to obtain greater safety and efficacy [3,4].

Acute inflammation has two main components: vascular changes and cellular events. The presence of infection or injury is perceived by resident cells, mainly macrophages, but also by other cell types, which secrete molecules (cytokines and other mediators) that induce and regulate the inflammatory response. Carrageenan (a polysaccharide) is a flogistic agent extensively used to induce an acute, non-immune, and highly reproducible inflammation framework in laboratory animals [5]. Sulfated sugars present in carrageenan are responsible for the activation of inflammatory mediators and the production of vascular and cellular events of inflammation [6].

Lectins are proteins that interact specifically and reversibly with carbohydrates and possess many biotechnological and biomedical applications [7,8]. They have been isolated from the most diverse sources, including plants. Plant lectins have shown a strong therapeutic potential [9] and their use has been suggested to combat pathologies, such as cancer [10,11]; bacterial [12], fungal [13], and viral [14] infections; and inflammatory conditions [15,16]. Lectins can also display an immunomodulatory action through interaction with oligosaccharides present on immune cell surfaces, triggering signal transduction for the modulation of cytokines, for example [17].

Plant lectins have shown an anti-inflammatory activity in vivo. *Canavalia boliviana* (Fabaceae) seed lectin (Cbol) was able to inhibit carrageenan-promoted paw edema and neutrophil migration in a peritonitis assay in Wistar rats [18]. The lectin isolated from *Mucuna pruriens* (Fabaceae) seeds (MPLEC) showed anti-edematogenic properties in the intense edema model caused by carrageenan in Swiss mice [19]. *Bauhinia bauhinioides* (Fabaceae) seed lectin (BBL) reduced carrageenan-induced paw edema and inhibited leukocyte migration and tumor necrosis factor (TNF-α) release in an experimental model of peritonitis in Wistar rats [20]. *Tetracarpidium conophorum* (Euphorbiaceae) seed lectin (TcSL) prevented leukocyte migration to the peritoneum and reduced carrageenan-induced paw edema in Wistar rats [5]. A seed lectin (ZOSL) from *Zizyphus oenoplia* (Rhamnaceae) showed an anti-inflammatory activity, preventing anaphylactic shock in Wistar rats [21].

*Microgramma vacciniifolia* (Polypodiaceae) frond lectin (MvFL) stimulated in vitro the production of TNF-α, interleukin (IL) 6, interferon-gamma (IFN-γ), and nitric oxide (NO) by human peripheral blood mononuclear cells (PBMCs), with a concomitant increase in the release of the regulatory cytokine IL-10. In addition, it induced the activation and differentiation of T CD8+ cells [22]. It has also been shown that MvFL (10 and 20 mg/kg) exhibits an antitumor activity against sarcoma 180 in mice, reducing tumor weight in 89–96% and interfering with the angiogenesis around the tumors; in addition, adverse effects were not seen in the animals treated with MvFL at both doses [23].

**Figure 1:**
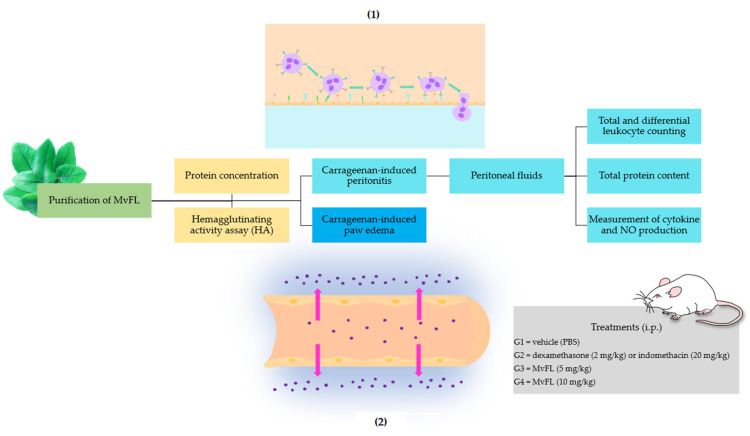
Graphical representation of the study design. The effects of MvFL on inflammatory status in the carrageenan-induced peritonitis and paw edema were investigated in Swiss mice. The animals were pretreated intraperitoneally with MvFL. In the peritonitis assay, the total and differential migration of the leukocytes were evaluated, as well as the total protein, cytokines, and nitric oxide (NO) levels in the peritoneal fluid. The inset (**1**) schematizes the recruitment of leukocytes to the area of inflammation. In the paw edema assay, the volume of the paw was measured in the early (30 min–2 h) and late (3–4 h) phases of edema formation. The inset (**2**) shows the edema formation process with extravasation of the plasma proteins followed by movement of the fluid from the intravascular to the interstitial space.

The previous report on the immunomodulatory activity of MvFL in vitro constituted evidence that this lectin can influence the release of inflammation mediators by immune cells. Thus, we hypothesized that this lectin could be an anti-inflammatory agent in vivo. To test this hypothesis, and then contribute to the search for new anti-inflammatory drugs, we used the experimental design summarized in Figure 1. Briefly, the effects of MvFL on two models of acute inflammation induced by carrageenan in Swiss mice were investigated. In the peritonitis assay, the migration of leukocytes and the presence of cytokines, NO, and total proteins in the peritoneal fluid were evaluated. In the paw edema model, we evaluated the anti-edematogenic action of MvFL.

## 2. Materials and Methods

### 2.1. Purification of MvFL

Fronds of *M. vacciniifolia* were collected at the campus of the *Universidade Federal de Pernambuco* (UFPE) at Recife, Brazil, with permission (72,024) from the *Instituto Chico Mendes de Conservação da Biodiversidade* (ICMBio, Brasília, Brazil). The project was registered (A9D147B) in the *Sistema Nacional de Gestão do Patrimônio Genético e do Conhecimento Tradicional Associado* (SisGen). Taxonomic certification was performed at the herbarium Dárdano de Andrade Lima (*Instituto Agronômico de Pernambuco*, Recife, Brazil), where an exsiccata (number 63,291) was filed.

The fronds were cleaned with a faucet and distilled water, dried at 28 °C for seven days, and then crushed using a blender. The proteins were extracted by intermixing the powder (10 g) in 150 mM NaCl (100 mL) for 16 h at 25 °C using a magnetic stirrer. After filtration over filter paper and centrifugation (9000× *g*, 4 °C; 15 min), the frond extract was obtained, which was used to isolate MvFL, as stated by Patriota et al. [22]. The purification procedure corresponded to the application of the extract (3.0 mg of protein) in a Sephadex G-75 (Sigma-Aldrich, St. Louis, MO, USA) column (30.0 cm × 1.5 cm) and subsequent loading (2.5 mg of protein) of the protein peak with hemagglutinating activity onto a DEAE-Sephadex A25 (GE Healthcare Life Sciences, Uppsala, Sweden) column (7.5 cm × 1.5 cm), earlier balanced with 100 mM Tris-HCl pH 8.0. MvFL was eluted from the column with 100 mM Tris-HCl pH 8.0 containing 1.0 M NaCl, dialyzed against distilled water for 4 h and lyophilized to complete dryness.

### 2.2. Protein Concentration

The protein concentration was predicted according to Lowry et al. [24], employing a standard curve of albumin from bovine serum (31.25–500 μg/mL). In this assay, the sample (0.2 mL) was incubated (10 min) with 1 mL of copper solution (1 mL of 0.5%, *w*/*v*, copper sulfate in 1%, *w*/*v*, sodium citrate plus 50 mL of 2%, *w*/*v*, sodium carbonate in 0.1%, *w*/*v*, sodium hydroxide). After incubation at 25 °C, 100 µL of the Folin–Ciocalteus reagent (Sigma-Aldrich), previously diluted 1:1 with distilled water, was added to the test tubes and, 30 min later, the absorbance at 720 nm was measured.

### 2.3. Hemagglutinating Activity Assay (HA)

The carbohydrate-interacting ability of MvFL was monitored by the hemagglutination assay [22] using rabbit red blood cells fixed with glutaraldehyde [25]. HA was calculated as the reciprocal value of the maximum sample dilution that still promoted cell agglutination. Specific HA was determined as the ratio between the HA and protein content (mg/mL).

### 2.4. Animals

The tests were developed at the *Laboratório de Oncologia Experimental* from the *Universidade Federal do Vale do São Francisco* (UNIVASF). Swiss females (*Mus musculus*) from the UNIVASF vivarium, aged between 6 and 8 weeks, were housed in a polypropylene box, at controlled temperature (24 °C) and 12-h light/dark cycles, with *ad libitum* access to water and aliment. Eight hours before the experiments, the animals were deprived of food, but unrestricted access to water was maintained.

### 2.5. Carrageenan-Induced Peritonitis

#### 2.5.1. Induction of Inflammation and Treatments

Swiss female mice were divided into four experimental groups of six animals each (*n* = 6). The leukocyte migration was stimulated by the inoculation of carrageenan (1%, 0.25 mL) into the intraperitoneal cavity 1 h after the administration of MvFL (5 and 10 mg/kg, i.p.), dexamethasone (2 mg/kg, i.p.), or the vehicle (phosphate buffered saline—PBS, i.p.). Dexamethasone (glucocorticoid) was chosen as a positive control because it possesses effects on both vascular and cellular events of the peritonitis model [6]. After 4 h of carrageenan inoculation, the animals were euthanized, and 3 mL of PBS containing 1 mM ethylenediaminetetraacetic acid (EDTA) was injected into the peritoneal cavity. Immediately, a massage was performed in the abdominal cavity and then the peritoneal fluid was collected with a 3 mL syringe and was centrifuged (3 000 rpm for 5 min) at 25 °C [26]. The supernatant was placed in a tube for the subsequent measurement of the cytokines, NO, and total proteins. The pellet was used for the total and differential leukocyte counting.

#### 2.5.2. Leukocyte Counting

The peritoneal fluid pellet was resuspended in 0.3 mL of PBS-EDTA solution; after homogenization, an aliquot of 10 μL was retrieved and 0.2 mL of Turk’s solution was added to it for further counting in a Neubauer chamber under an optical microscope [27]. The data were expressed as the number of white blood cells per mL. The leukocyte inhibition (%) was calculated as (1 − T/C) × 100, where T corresponds to the leukocyte counts for the treated groups and C is the leukocyte count in the negative control (vehicle) [28]. The differential evaluation of leukocytes was accomplished under an optical microscope, after staining in a Giemsa’s solution (10%) of smears obtained after centrifuging the peritoneal liquid, being 100 cells counted per slide [29].

#### 2.5.3. Total Protein Content

Fluids retrieved from the peritoneal cavity of the mice treated with MvFL, dexamethasone, or vehicle (PBS) after carrageenan-induced peritonitis were centrifuged (3000 rpm for 5 min) and the total protein content was assessed in the supernatant according to Lowry et al. [24], as presented in Section 2.2. The results obtained in mg/mL were multiplied by the volume of peritoneal fluid collected (3 mL).

#### 2.5.4. Measurement of Cytokine and NO Production

Supernatant of peritoneal fluids obtained from animals treated with MvFL, dexamethasone or vehicle (PBS) after carrageenan-induced peritonitis were collected for cytokine quantification using the Cytometric Bead Array (CBA) Mouse Th1/Th2/Th17 Cytokine Kit (Becton Dickinson Biosciences, Franklin Lakes, USA) for concomitant detection of interleukins (IL-2, IL-4, IL-6, IL-10, and IL-17A), interferon-gamma (IFN-γ), and TNF-α. The evaluations were performed following the manufacturer’s instructions and data were obtained in the BDAccuri C6 (BD Biosciences) cytometer. Seven individual cytokine standard curves (20–5000 pg/mL) were achieved. The results were analyzed using the BDAccuri C6 software (BD Biosciences).

NO production was measured with the Griess method [30]. The NO concentration was estimated using a standard curve of nitrite (3.12–400 µmol/mL) and a spectrophotometer for microplates (Thermo Fisher Scientific) at 595 nm.

### 2.6. Carrageenan-Induced Paw Edema Assay

Paw edema was induced by injecting 20 μL of 1% carrageenan into the subplantar region in the right hind paw of each mouse. The animals were separated into four experimental groups of six mice each (*n* = 6) and treated with MvFL (5 and 10 mg/kg, i.p.), indomethacin (20 mg/kg, i.p.) or vehicle (PBS, i.p.) 1 h before the injection of carrageenan. Indomethacin (non-steroidal anti-inflammatory drug—NSAID) is a prostaglandin antagonist that functions as an excellent positive control in the paw edema model because it inhibits vascular events [6]. The volume of the paw was recorded using a plethysmometer (PanLab LE 7500, Spain) by submerging the animals paws up to the lateral malleolus of the heel [26]. Measurements were taken before carrageenan inoculation and 1, 2, 3, and 4 h after [27].

### 2.7. Statistical Analysis

The data were expressed as means of replicates ± standard error of the mean (SEM), which were calculated using GraphPad Prism 5 (GraphPad Software, San Diego, CA, USA). Significant differences were determined using one-way analysis of variance (ANOVA) followed by Tukey’s test employing the same program.

## 3. Results

**Figure 2:**
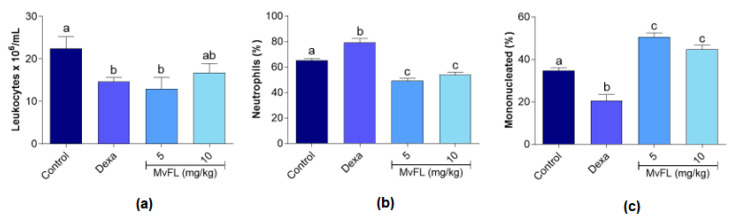
Effects of MvFL (5 and 10 mg/kg) and dexamethasone (2 mg/kg) on leukocyte migration in the peritoneal cavity of mice submitted to carrageenan-induced peritonitis. The graphs show the number of leukocytes per mL (**a**) and the percentage of neutrophils (**b**) and mononucleated cells (**c**) among them. Values are expressed as mean ± standard error of the mean (*n* = 6). Different letters above the bars indicate significant (*p* < 0.05) differences between treatments according to ANOVA followed by Tukey’s test.

MvFL was successfully purified, showing a specific hemagglutinating activity of 10,240, which confirms that its carbohydrate-interacting property was working. In the peritonitis assay, the number of total leukocytes in the peritoneal cavity was significantly (*p* < 0.05) reduced in the treatment with MvFL at 5 mg/kg (leukocyte inhibition of 42.4%) when compared with negative control (Figure 2a). The effect was not statistically significantly (*p* > 0.05) different from that observed for dexamethasone (34.5%). At the dose of 10 mg/kg, the MvFL did not significantly reduce (*p* > 0.05) the number of total leukocytes in comparison with the negative control.

When the differential leukocyte evaluation was performed, it was observed that treatments with MvFL at both doses significantly (*p* < 0.05) reduced the percentage of neutrophils in the peritoneal fluid from 65.2% in the control to 49.25% (5 mg/kg) and 54.0% (10 mg/kg) Figure 2b). Lectin treatment also increased the percentage of mononucleated cells to 50.5 (5 mg/kg) and 44.7% (10 mg/g) (Figure 2c), especially lymphocytes, when compared to the negative control (34.7%). In contrast, dexamethasone treatment increased the proportion of neutrophils and decreased that of the mononucleated cells (Figure 2b,c, respectively).

**Figure 3:**
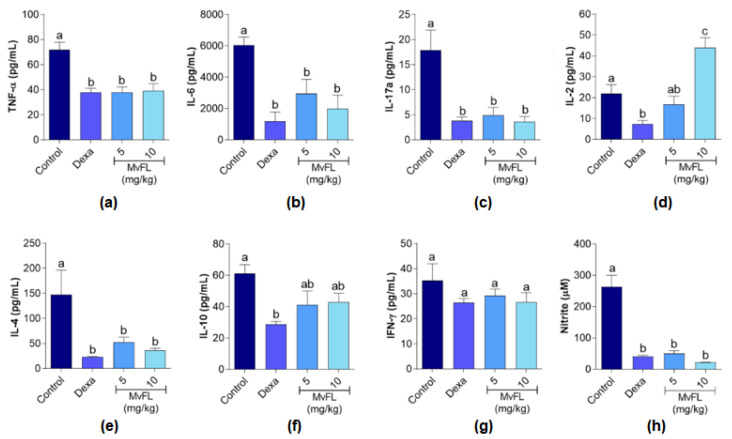
Cytokine (**a**–**g**) and nitrite (**h**) concentrations in the peritoneal fluid of mice submitted to carrageenan-induced peritonitis after pre-treatment with MvFL (5 and 10 mg/kg) or dexamethasone (2 mg/kg). Values are expressed as mean ± standard error of the mean (*n* = 6). Different letters above the bars indicate significant (*p* < 0.05) differences between treatments according to ANOVA followed by Tukey’s test.

The assessment of cytokine levels (Figure 3) revealed reduced (*p* < 0.05) release of IL-4, IL-6, IL-17A, and TNF-α in the peritoneal fluid of mice treated with MvFL at both doses (5 and 10 mg/kg) or dexamethasone, when compared to the negative control. The levels of IL-10 (Figure 3f) and IFN-γ (Figure 3g) did not differ significantly (*p* > 0.05) from the negative control, while the IL-2 level (Figure 3d) was higher in the group that received MvFL at 10 mg/kg. Treatments with MvFL (5 and 10 mg/kg) also significantly reduced (*p* < 0.05) the levels of NO (Figure 3h) when compared with the negative control, as it was observed for the group treated with dexamethasone.

**Figure 4:**
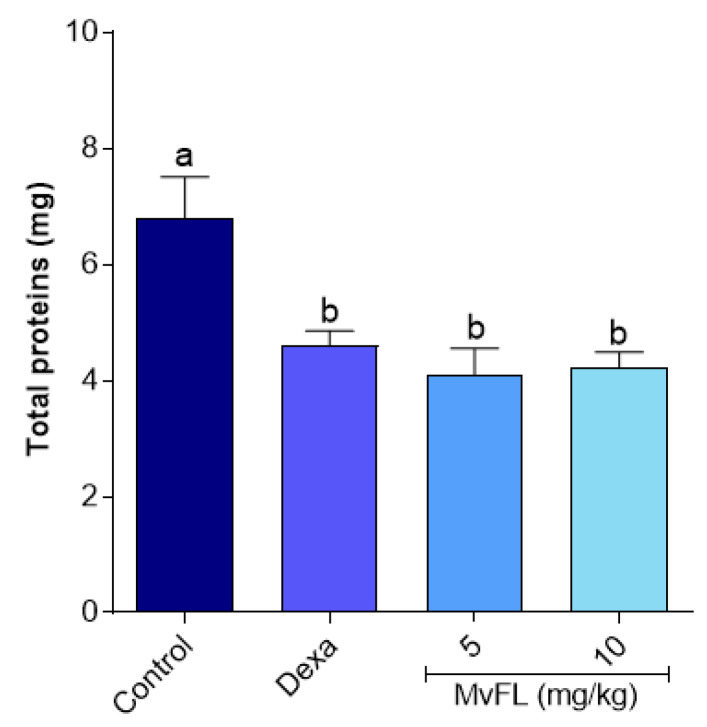
Total protein concentration in the peritoneal liquid from mice submitted to carrageenan-promoted peritonitis after pre-treatment with MvFL (5 and 10 mg/kg) or dexamethasone (2 mg/kg). Values are expressed as mean ± standard error of the mean (*n* = 6). Different letters indicate significant (*p* < 0.05) differences between treatments according to ANOVA followed by Tukey’s test.

Plasma leakage into the peritoneal cavity was indirectly assessed by measuring the total protein concentration in the peritoneal liquid. The results indicate that MvFL (5 and 10 mg/kg) or dexamethasone significantly reduced (*p* < 0.05) the inflammatory exudate (Figure 4) by 39.5%, 37.6%, and 30.1%, respectively, when compared to the negative control.

**Figure 5:**
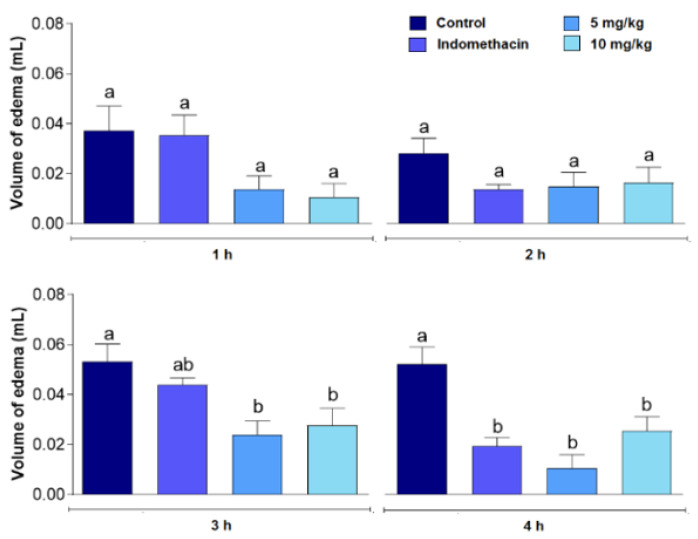
Anti-inflammatory effect of MvFL (5 and 10 mg/kg) and indomethacin (20 mg/kg) on carrageenan-promoted paw edema. Values are expressed as mean ± standard error of the mean (*n* = 6). Different letters indicate significant (*p* < 0.05) differences between treatments according to ANOVA followed by Tukey’s test.

The results of the paw edema assay can be seen in Figure 5. MvFL did not significantly inhibit (*p* > 0.05) the formation of the edema in the early phase (1 and 2 h) at any of the doses used in the assays, similarly to the indomethacin-treated group. On the other hand, a significant (*p* < 0.05) edema reduction in groups treated with MvFL at both doses was observed after 3 h and 4 h, while the group treated with indomethacin showed significant inhibition after only 4 h.

## 4. Discussion

The peritoneum functions as a hub for leukocytes, which readily populate the site as a consequence of an induction of inflammation; thus, the peritonitis model induced by carrageenan has been widely used [21,31,32,33]. The inflammation produced by this model makes it possible to assess the cell migration, participation of cytokines and chemical mediators, and protein extravasation. Although MvFL and dexamethasone both reduced the number of leukocytes in the peritoneal fluid, the fact that neutrophils were the majority among the leukocytes in the dexamethasone-treated group, while monocytes were mostly found in fluids from lectin-treated animals, suggests that these molecules have distinct mechanisms for modulating the role of immune cells in the resolution of inflammation. As already mentioned, MvFL is capable of acting on human mononuclear cells [22], which seems to also occur on mice cells. The reduced proportion of neutrophils as well as the lower levels of TNF-α, IL-6, IL-17A, IL-4, NO, and total proteins in the peritoneal fluid confirm that this lectin could influence the concentration of inflammation mediators released by immune cells.

The cytokine profile in the culture supernatant of the MvFL-treated human PBMCs [22] was different from that observed in the peritoneal fluid of the MvFL-treated animals reported here. Indeed, the effects of immunomodulatory agents can vary depending on the assay conditions and experimental models. There is a more complex network of interactions between cells and organs in in vivo conditions, and the profile of modulated cytokines depends on the concentration and route of administration [34]. In addition, there was no inflammatory condition when the immunomodulatory activity of MvFL was evaluated on PBMCs. Together, the data reveal that distinct modulation profiles can be generated by MvFL at in vitro or in vivo conditions, as well as when an inflammatory condition is present or not.

Similar to MvFL at 5 mg/kg, *Lonchocarpus araripensis* (Fabaceae) seed lectin (LAL) (10 mg/kg) reduced the leukocyte migration induced by carrageenan in male Wistar rats by 43% [16], and the lectin from *Bauhinia monandra* (Fabaceae) leaves (BmoLL) (15, 30, and 60 mg/kg) significantly reduced (43.5, 54.9, and 60.9%, respectively) leukocyte migration into the peritoneum of male Swiss mice [32]. More details on the similarities and differences in the findings for these three lectins are discussed below.

The reduced percentage of neutrophils and increased percentage of mononucleated cells detected in the treatment with MvFL may be indicative that this lectin favors the lymphocyte response. This result agrees with that found in a previous study, where MvFL promoted the activation and differentiation of human T CD8+ lymphocytes [23]. In a similar way, *Tetracarpidium conophorum* seed lectin (TcSL) (3, 6, and 12 mg/kg) reduced leukocyte migration into the peritoneum in Wistar rats, and a larger percentage of lymphocytes was found in the peritoneal fluid; the authors considered that treatment with TcSL accelerated the inflammation resolution, as lymphocytes participate in the process at the late phase of the inflammatory response [5]. In addition, similarly to MvFL and TcSL, LAL (10 mg/kg) reduced leukocyte migration with a reduction in the proportion of neutrophils [33]. LAL also inhibited the rolling and adhesion of white blood cells induced by carrageenan in male Wistar rats [16].

The decrease in TNF-α and IL-6 levels in the groups treated with MvFL confirms their anti-inflammatory action, as TNF-α is an important mediator of the acute inflammatory response and IL-6 is also important in acute inflammatory responses that have both local and systemic effects. These results can also be linked to the lower proportion of neutrophils, because TNF-α and IL-6 produced at inflammatory sites can enter the blood and be distributed to the bone marrow, where they increase the production of neutrophils from the bone marrow progenitors, usually acting in conjunction with colony stimulating factors [33].

IL-17A has an essential role in the acute inflammation and has been reported to contribute to the proinflammatory environment by prompting the release of IL-6 and TNF-α. In addition, IL-6 also provokes the differentiation of IL-17-producing T cells [33], and thus it is understandable that IL-6, TNF-α, and IL-17A levels decreased in the treatments with MvFL. Anti-IL-17A therapy has been employed in the treatment of patients suffering from inflammation [35,36].

Interestingly, the IL-2 level in animals treated with MvFL at 10 mg/kg increased when compared to the negative control. IL-2 is the main factor stimulating the growth and activation of lymphocytes [37], which were found to be the most abundant cell population that migrated to the inflammatory site after treatment with MvFL. It has been previously described that MvFL is capable of eliciting the differentiation and activation of T CD8+ lymphocytes in vitro, as determined by an immunophenotyping assay for the labeling of cells expressing the CD28 protein [22].

Immunosuppressive cytokines, including IL-4 and IL-10, are typically produced at peripheral sites in a delayed manner to reduce the level of pro-inflammatory cytokine production by activated cells and to promote tissue repair [38]. As treatments with MvFL reduced IL-4 levels and did not affect IL-10 levels, its anti-inflammatory action seems to be mainly related to a reduction in the levels of proinflammatory cytokines with maintenance of the levels of IL-10 regulatory cytokine.

The modulation of cytokine levels by plant lectins in models of acute inflammation has been described. For example, *Parkia biglobosa* (Fabaceae) seed lectin (PBL) induced the release of anti-inflammatory cytokines in a carrageenan-induced peritonitis model [39]. On the other hand, *Lonchocarpus sericeus* (Fabaceae) seed lectin (LSL) decreased the leukocyte migration and inflammatory response in male Balb/c mice via the inhibition of pro-inflammatory cytokines (TNF-α and IL-1β) [40], which is similar to what was detected in the treatments with MvFL.

The ability of MvFL to strongly reduce NO levels is advantageous in the anti-inflammatory context, because NO can rapidly react with free radicals such as superoxide anion (O_2_^−^•) to produce the highly reactive oxidant peroxinitrite (ONOO−) and other reactive nitrogen species (RNS), which are linked to pathological conditions (such as chronic inflammation, autoimmune diseases, and cancer), amplifying inflammatory circuits [41]. In addition, the inhibition of NO release can be due to the down-regulation of iNOS as TNF-α induces NO synthesis by activating iNOS [42].

During acute inflammation, vasodilation and increased vascular permeability are induced by chemical mediators such as histamine, allowing leukocytes and plasma proteins to enter the extravascular tissues. This causes an increase in the osmotic pressure of the interstitial fluid, leading to a greater efflux of water from the blood to the tissues. The resulting accumulation of protein-rich fluid (exudate) in the extravascular spaces is called edema [43,44]. Carrageenan-promoted paw edema is a well-established model of acute inflammation and has been widely used to evaluate the anti-edematous effect of natural products [15,18]. In mice, carrageenan subplantar injection provokes a biphasic edema, which is then divided into early phase (from 30 min–2 h) and late phase (3–4 h). The early phase is mainly due to the action of vasoactive amines: histamine, serotonin, and bradykinin, which increase vascular permeability [26] and are released from mastocytes into the circumambient of damaged tissues [45]. The late phase is sustained by prostaglandins, leukotrienes, and cytokines such as IL-6 and TNF-α, produced by tissue macrophages and polymorphonuclear cells [45,46].

Considering that MvFL reduced leukocyte migration, pro-inflammatory cytokines, NO, and protein leakage in the peritonitis model, we evaluated the anti-edematogenic effect of MvFL in the carrageenan-promoted paw edema model. The results suggest that MvFL acts in the second phase (3–4 h) of edema development, probably due to the ability to cause a decrease in the levels of pro-inflammatory cytokines and chemical mediators such as NO, which are important in maintaining this phase of edema.

On the other hand, *Lonchocarpus campestris* (Fabaceae) seed lectin (LCaL) (10 mg/kg) inhibited the edema induced by carrageenan in male Swiss mice, mainly in the first phase [15], and TcSL (3 mg/kg, 6 mg/kg, 12 mg/kg) reduced the Wistar rats paw edema in a dose-dependent way, with the animals that received the dose of 12 mg/kg showing a remarkable minimal edema in the second hour [5]. Otherwise, and similarly to MvFL, LAL (0.1 and 1 mg/kg) reduced the paw edema−time course induced by carrageenan in male Wistar rats, mainly in the second phase [16]. In the same way, BmoLL (30 and 60 mg/kg) showed an anti-inflammatory activity by decreasing the paw edema in a dose-dependent fashion in male Swiss mice, with the most significant paw edema reduction being observed after 4 h [32].

## 5. Conclusions

The present study demonstrated that MvFL, when intraperitoneally administered, has an anti-inflammatory effect in two models of acute inflammation, influencing the release of inflammation mediators by immune cells. MvFL treatment was capable of reduce neutrophil infiltration, favoring the lymphocyte response, and decrease the levels of pro-inflammatory cytokines and NO in the peritoneal fluid. In addition, the maintenance of the levels of IL-10 was an interesting characteristic of the modulating profile promoted by MvFL. Thus, it is highlighted that the ability of MvFL to modulate all these mediators can be used therapeutically to control, regulate, and limit acute inflammatory processes to prevent damage. The results stimulate more studies on the effects of MvFL on animal models of inflammatory diseases such as asthma. In addition, studies on the molecular interactions that trigger these MvFL effects are now necessary to understand in detail its action mechanisms. To our knowledge, MvFL is the single protein isolated from plants of this family that showed immunomodulatory properties.

## Data Availability

The raw data presented in this study are available upon request from the corresponding author. The data are not publicly available due to privacy restrictions.
